# Identifying and tracking mobile elements in evolving compost communities yields insights into the nanobiome

**DOI:** 10.1038/s43705-023-00294-w

**Published:** 2023-08-28

**Authors:** Bram van Dijk, Pauline Buffard, Andrew D. Farr, Franz Giersdorf, Jeroen Meijer, Bas E. Dutilh, Paul B. Rainey

**Affiliations:** 1 Department of Microbial Population Biology, Max Planck Institute for Evolutionary Biology, Plön, Germany; 2 Theoretical Biology and Bioinformatics, Department of Biology, Science for Life, Utrecht University, Utrecht, the Netherlands; 3 Institute of Biodiversity, Faculty of Biological Sciences, Cluster of Excellence Balance of the Microverse, Friedrich Schiller University, Jena, Germany; 4 Laboratory of Biophysics and Evolution, CBI, ESPCI Paris, Université PSL CNRS, Paris, France

**Keywords:** Metagenomics, Microbial genetics, Molecular evolution

## Abstract

Microbial evolution is driven by rapid changes in gene content mediated by horizontal gene transfer (HGT). While mobile genetic elements (MGEs) are important drivers of gene flux, the nanobiome—the zoo of Darwinian replicators that depend on microbial hosts—remains poorly characterised. New approaches are necessary to increase our understanding beyond MGEs shaping individual populations, towards their impacts on complex microbial communities. A bioinformatic pipeline (xenoseq) was developed to cross-compare metagenomic samples from microbial consortia evolving in parallel, aimed at identifying MGE dissemination, which was applied to compost communities which underwent periodic mixing of MGEs. We show that xenoseq can distinguish movement of MGEs from demographic changes in community composition that otherwise confounds identification, and furthermore demonstrate the discovery of various unexpected entities. Of particular interest was a nanobacterium of the candidate phylum radiation (CPR) which is closely related to a species identified in groundwater ecosystems (Candidatus Saccharibacterium), and appears to have a parasitic lifestyle. We also highlight another prolific mobile element, a 313 kb plasmid hosted by a *Cellvibrio* lineage. The host was predicted to be capable of nitrogen fixation, and acquisition of the plasmid coincides with increased ammonia production. Taken together, our data show that new experimental strategies combined with bioinformatic analyses of metagenomic data stand to provide insight into the nanobiome as a driver of microbial community evolution.

## Introduction

Horizontal gene transfer (HGT) can markedly affect the evolutionary fate of microbes [1–3]. Besides transformation—where bacteria directly take up environmental DNA—all horizontal movement of genetic material is catalysed by mobile genetic elements (MGEs), Darwinian entities with dynamics of their own [4]. Last century, a multiplicity of MGEs has been observed, ranging from the distinctly parasitic bacteriophages [5–9] and transposons [10–12], to plasmids [13–15], and integrative and conjugative elements (ICEs) [16–19]. More recently, new mobile elements are being discovered all across the microbial world, such as REPINs [20, 21], Starships [22], and Borgs [23], and even entire fungal chromosomes appear to be on the move [24–26].

The relationship between MGEs and hosts are complex, ever changing, and highly context-dependent. For example, while conjugative elements are typically benign, they may also promote self-survival at the expense of hosts [19, 27]. Similarly, while bacteriophages are typically predatory or parasitic, they can be co-opted to benefit hosts [5, 28–30]. Moreover, MGEs may recombine with one another or parasitise other mobile elements [31–35]. Taken together, these processes may be fundamental to our understanding of microbial communities as collections of locally adaptive genes, rather than locally adapted species [36, 37]. While the scope and scale of DNA flux through microbial communities via MGEs is currently poorly understood, recent work suggests that the flux may be highly significant even to the extent that it defines and drives a community-level process with effects similar to sex within populations [38, 39].

In addition to moving genes necessary for self-replication and transmission, MGEs often mediate transfer of host genes to which they become linked. Whether by design, or accident, MGEs that acquire genes that enhance host fitness stand to be rapidly amplified by selection with captured genes being widely disseminated. On occasion the effects can be highly consequential, for example, movement of ICEs carrying genes for nodulation and nitrogen fixation convert non-symbiotic rhizobia into plant symbionts in a single step [40, 41]. ICEs have also been identified by observing the movement of antimicrobial resistance [42, 43], and heavy metal resistance genes [18]. However, such routes to discovery depend on both abilities to culture focal microbes and carriage of selectable phenotypic traits.

With increasing ability to sequence complex communities, discovery of MGEs has been fuelled by metagenomics through culture-independent assembly of DNA replicons, without prior assumptions about biological relevance. Moreover, bioinformatic tools have been developed that can separate a wide range of candidate MGEs from microbial hosts [44–51]. Although such tools allow differentiation of chromosomal DNA from phages, plasmids, and other MGEs, discovery of MGEs is constrained by existing databases and training sets that are based on known MGEs. Moreover, metagenomic detection of MGEs *per se* rarely yields insight into the ecological relevance of the element. Unbiased detection and characterisation of unknown MGEs remains a major challenge.

Recently, Quistad et al. reported a generally applicable experimental strategy that connects movement of genes via MGEs to the ecology and functioning of complex microbial communities [52]. The strategy exploits the fact that long-term persistence of MGEs—and especially those that are costly to hosts—depends on horizontal transmission [53–55]. In the absence of opportunity to encounter new hosts, MGEs lose replicative capacity, resulting in either extinction, or co-option as permanent components of the host genome, at least in cases where elements encode host-beneficial fitness effects. Frequent exposure to new hosts, may instead breath ‘evolutionary life’ into MGEs, fuelling the continued co-evolution of MGEs and their hosts [13, 14, 56].

**Fig. 1:**
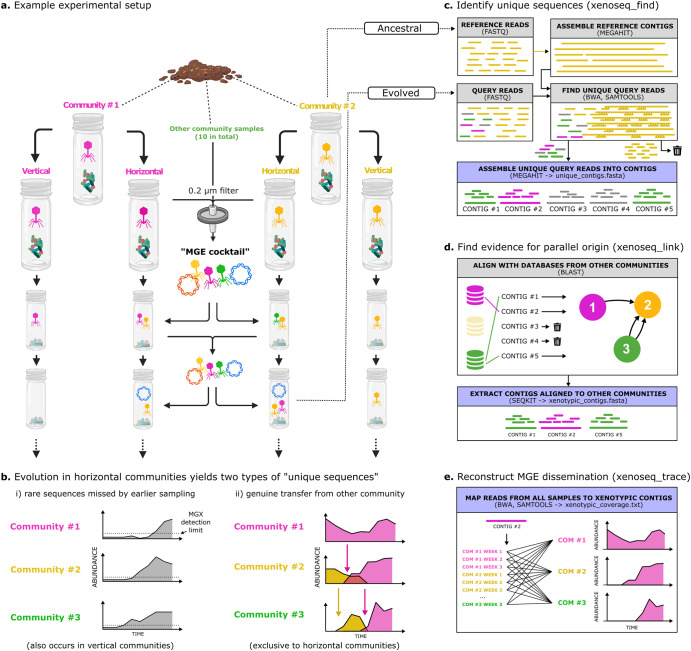
Experimental design and overview of bioinformatic pipeline. Overview of (**a**) experimental setup, (**b**) sources of unique sequences, and (**c**–**e)** the bioinformatic pipeline. **a** Experimental evolution protocol to identify novel MGEs and other mobile sequences [52]. Multiple parallel communities were established from garden compost, which were then propagated on cellulose as the sole carbon source and serially transferred every two weeks. Horizontal treatments were provided with an ‘MGE cocktail’ which was derived by pooling material collected from samples passaged through 0.2 µm filters. This manipulation allows MGEs and linked genes to transfer horizontally from one community to the next. **b** Sequencing samples obtained from mesocosms yields two types of ‘unique sequences’. Shown are cartoons to visualise how some unique sequences (left) are the result of rare sequences that were undetected by earlier sampling, while other unique sequences (right) are the result of genuine transfer from another community. **c** The first subroutine of xenoseq (xenoseq*_*find) compares raw DNA sequences reads in fastq format from one sample (query, hereafter called *evolved*) with another sample (subject, hereafter *ancestral*). Ancestral samples are assembled into contigs, which are used as bait to remove reads from the evolved samples. Unmapped reads from the evolved samples are then assembled into ‘unique contigs’, stretches of DNA that have newly appeared in the community. **d** To distinguish between two sources of unique sequence shown in panel b, the second subroutine (xenoseq_link) identifies candidate mobile elements by aligning them to all other ancestral communities. The subset of contigs that align to allopatric communities are referred to as *xenotypic contigs*. **e** Finally, the dissemination patterns of xenotypic contigs is reconstructed by mapping DNA sequences reads from all communities against assembled contigs. Sequencing depth and breadth are stored in a tabular text file.

In the study by Quistad et al., garden compost communities were established and maintained for 48 weeks in glass mesocosms. These communities were further split into two pairs: horizontal- and vertical communities. For vertical communities (hereafter: V communities), a sample of the community (including MGEs) is periodically transferred to a fresh mesocosm. Horizontal communities (hereafter: H communities) are treated similarly, but with one important difference: at the time of transfer, a sample from all independent H communities is passed through a 0.2 µm filter and the filtrate collected. Filtration removes bacteria and larger entities, but allows collection of material smaller that 0.2 µm that stands to include recognised MGEs such as phages, but also yet-to-be-discovered elements, and additionally naked DNA, minerals, nutrients and so forth. This filtrate is referred to as an ‘MGE-cocktail’. The combined MGE cocktail of all independent communities is then redistributed across all H communities (see Fig. 1a). Evolved communities were then subjected to metagenomic sequencing, which—by virtue of the experimental design—allowed the identification of sequences in the H communities that were not detected in the respective ancestral communities (hereafter referred to as ‘unique sequences’). While their initial detection was not dependent on matches to existing viral databases, many of these unique sequences were shown to indeed encode phage-related proteins. In principle, however, any element in the MGE-cocktail with the ability to amplify after introduction into a new horizontal community, could be detected with this experimental design.

Here, we describe a broadly applicable ‘xenoseq’ pipeline (github.com/bramvandijk88/xenoseq) that takes as input, raw sequencing reads from time series experiments, and identifies sequences that have been transferred from allopatric communities and amplified by replication in the current community. When applied to metagenomic data from the experiment performed by Quistad et al., we show that xenoseq readily detects candidate MGEs whose dynamics (including community of origin) can be followed through time. One important attribute of xenoseq is ability to distinguish between sequences that are selected due to demographic changes in patterns of species abundance, and those that are horizontally disseminated from an allopatric community. The latter are hereafter referred to as ‘xenotypic sequences’. We show that xenotypic sequences are enriched in recognisable components of phages and IS-elements, that is, canonical selfish genetic elements (SGEs). Less expected was the observation of horizontally transmitted nanobacteria and large plasmids. To explore the dynamics of MGEs through the course of the year-long study, Metagenome-Assembled Genomes (MAGs) were constructed by cross assembly, providing insights into the potential hosts of these unexpected elements, and allowing the dissemination of MGEs to be linked to the community-level changes in ammonia production rates.

## Materials and methods

### Bioinformatic pipeline to detect transfer events

The xenoseq pipeline (https://github.com/bramvandijk88/xenoseq, Fig. 1c–e) is a wrapper that combines read trimming, assembly, read mapping, read filtering, and local alignment to seek evidence of horizontal gene transfer or the transfer of other nanoscale entities between evolving communities. This pipeline takes as input raw (untrimmed) fastq files containing shotgun metagenomic data of derived samples (query, in which to search for newly introduced sequences) and datasets from ancestral samples (reference). Query reads are trimmed using fastp [57] (v0.23.2), mapped against the corresponding reference contigs using BWA [58, 59] (v0.7.17) using default options, and samtools [60] (v1.15.1) is used with the flag ‘−*4’* to extract unmapped reads. These reads are assembled into ‘unique contigs’ using Megahit [61] (v1.2.9) (*xenoseq_find*, Fig. 1c). Next, these unique contigs are blasted against a local database using NCBI Blast [62] (v.2.13.0) of all other reference communities to link the emergence of unique contigs to allopatric ‘donor’ communities. Linked contigs are extracted from unique contigs using Seqkit [63] (v.2.3.0) (*xenoseq_link*, Fig. 1d). By default, contigs are linked to a donor when blast has at least one high-scoring segment with a minimum length of 300 with at least 99% nucleotide identity. While the algorithm and cut-offs limit the sensitivity over larger evolutionary distances, they are designed to detect sequences that have recently diverged and are still largely identical. The contigs that match target sequences in allopatric communities are referred to as ‘*xenotypic contigs*’. Finally, to detect shifts in abundance of the detected contigs, reads from all query and reference samples are mapped (*xenoseq_trace*), and coverage/breadth statistics are saved in a tab-separated file. This allows for visualisation of transfer across communities (Fig. 1e). In this manuscript, we further analyse sequences >10 kb in length.

By default, xenoseq runs all subroutines (*xenoseq_find, xenoseq_link, xenoseq_trace*), but each subroutine can be run independently by modifying the command-line flags. Finally, xenoseq uses GNU parallel^59^ to run multiple jobs simultaneously.

### Xenoseq benchmark

To benchmark the pipeline, introduction of MGEs into mock-communities was simulated (see Supplementary Material I full details). Six bacterial genome sequences were downloaded from RefSeq. Two mock-communities were then generated, one with an even taxon distribution (easy dataset), and one with a highly skewed taxon distribution (hard dataset). Simulated MGE sequences were either i) randomly inserted into genome sequences, representing integrative elements, or ii) included as separate replicons linked to a single host genome by addition to the genome fasta file as a separate contig. Illumina sequencing was simulated from the resulting ‘communities’ with ART^62^, using both default and ten-fold elevated error rates, which were used as input to benchmark the pipeline. Simulation of mock-communities and horizontal gene transfer events was done in R v4.1.3 using the packages biostrings^60^ v2.62.0 and seqinr^61^ v4.2.16. Another (small) mock-community dataset is available on the repository, and can be used to rapidly test whether the pipeline and its dependencies are configured correctly.

### Metagenome-assembled genomes

We generated MAGs from all twenty compost communities. By combining samples across multiple time points, we improved the potential detection of rare types whose coverage in a single sample is insufficient for assembly. Quality control of sequencing reads was done with Prinseq version 0.20.4 [64] using *‘–derep 14 -lc method -c threshold 20’*. We trimmed adapters with Flexbar v.3.5.0 [65] using the ‘*–adapter-preset Nextera -ap ON*’ flag. Combined reads from multiple time points for each community were cross-assembled *de novo* using metaSPAdes v.3.14.0 [66]. Reads from each sample were mapped back to the assembled contigs using bwa-mem v.0.7.17-r1188 [59], and coverage calculations were performed with SAMtools v1.7 [60]. Contigs were binned using metaBAT2 v2.12.1 [67] and MAG quality was assessed with CheckM v1.1.3 [68]. All MAGs and contigs were annotated using BAT and CAT (Bin/Contig Annotation Tool, respectively, v.5.1.2) [69], which uses prodigal [70] to predict open reading frames and diamond [71] to query open reading frames against the NR database [72]. Where BAT or CAT were unable to reliably assign a taxon due to conflicting annotations between ORFs, a higher order taxonomic rank was assigned to the MAG: that is, to genus level if species identity could not be assigned, family level if genus could not be assigned, etc. CAT was called using parameters *‘--index_chunk 1 --block_size 5 --top 11’* using the CAT database and taxonomy constructed on 2021-04-30. Read mapping of samples from individual time points (above) was used to study the relative abundance of individual MAGs. The nanobacterium MAG was placed into a 16S phylogeny with MAGs from groundwater ecosystems from He et al. [73] by extracting 16S sequences with Barnapp 0.9, and a neigbour-joining tree was created with Geneious 2023.1.2.

### DNA degradation assay

To investigate whether the identified sequences are derived from living cells or DNA liberated by lysed cells, we tested the ability of microbial compost communities to degrade extracellular DNA. To do this, four experimental microbial communities were established from samples of four independent compost heaps. Compost was washed in M9 salt solution and stored with glycerol saline at −80 °C. Frozen stocks were thawed on ice and 4.25 mL of the stock was then washed of glycerol by two successive cycles of centrifugation (4000 *g*, 10 min) and resuspension (in 5 mL sterile M9 salt solution), followed by a final resuspension in 1 mL M9 salt solution. The washed cells were then added to 100 mL bottles with 19 mL M9 media and a piece of cellulose paper as a carbon source. Mesocosms were incubated without shaking at 28 °C for 14 days to allow community growth. The entire volume including any remaining paper was then transferred to 50 mL centrifuge tubes, vortexed for five minutes to produce a slurry, and 1 mL of slurry was transferred to fresh M9 media for another 14 days of incubation. During this time a stock of genomic DNA (gDNA) from *Escherichia coli* (REL606) was made using the ‘DNeasy Ultraclean Microbial kit’ (Qiagen), resulting in a stock of ~30 mL of gDNA at 20 µg/mL (stored at −20 °C).

Communities were then propagated a second time to four replicate cellulose-mesocosms spiked with gDNA. Community-free mesocosms were also established which acted as controls. All mesocosms were spiked with ten µg of *E. coli* gDNA, and were destructively harvested after 0, 1, 2 and 14 days of incubation. For sampling, the contents of each mesocosm was transferred to a 20 mL centrifuge tube, reduced to a slurry and then centrifuged at 4000 *g* for 5 min. The supernatant was transferred to 15 mL sterile tubes and frozen at −80 °C for later DNA extraction. In total, four replicate communities and three community-free controls were harvested at each time-point.

To measure relative DNA degradation by communities over the time-course, supernatant samples were thawed on ice, and further spiked with an internal standard of one µg/mL of gDNA of *Pseudomonas fluorescens* SBW25. Samples were then vortexed and samples were filtered using a 0.2 µm syringe-filter. DNA of 2 mL of spiked filtrate was isolated using a Phage DNA Isolation Kit (Norgen Biotek Corp) following the manufacturer’s instructions, except for loading two (rather than one) mL sample through a single purification column to improve DNA yield. Extracted DNA was then sequenced using a NextSeq MidOutput 300 cycle run resulting in 2 × 150 bp paired-end reads (see Supplementary Table I). Reads were aligned to the non-redundant (NR) protein database [72] with Diamond^54^ using the blastx subroutine, where the top hit was used to identify the read as being either *P. fluorescens*, *E. coli*, or ‘other’ (the latter representing either species present in the communities or erroneously assigned reads).

### MGE classification

Viral sequences were predicted using Vibrant (v1.2.0) [44], Virsorter2 (v2.2.3) [45], Seeker (v1.0.3) [46], and CheckV (v0.8.1) [74]. While CheckV is primarily designed to estimate completeness of candidate viral genomes, we found that sequences with either low, medium or high scores were typically identified as viral by other tools as well (see Fig. 3 in main text). Plasmid sequences were predicted using PlasFlow (v1.1.0)w [47] and PlasClass (v0.1) [48] and, if relevant, the origin of replication with OriFinder 2022 [75]. ICEs were identified with ICEfinder [49]. IS elements were predicted with ISEscan (v1.7.2.3) [51]. Integrons were predicted using Integronfinder2 (v2.0rc6) [50]. All open reading frames on MGEs were annotated with prokka [76] with both the default database and the PHROG database [77] for identification of viral genes. All tools were run with default options, results are shown in Supplementary Table 1, Sheet 3.

### MAG metabolic functions

The limiting resources in the M9 medium of the experiment by Quistad et al. are carbon (provided by the cellulose paper) and nitrogen (1 mM ammonium chloride from M9 salts), both of which are added every 14 days during serial passaging. To obtain insight into community function, MAGs were interrogated for the presence of genes predicted to be involved in cellulose degradation and nitrogen metabolism. Although many relevant proteins are involved, we focus on endoglucanases and cellobiosidases (cellulose degradation), nitrogenases (nitrogen fixation), and nitrate/nitrite reductase (nitrogen reduction). More ‘private’ functions, such as the ability to take up glucose, glycolysis, and downstream pathways, are not taken into consideration for this study. Protein sequences were extracted from the Uniprot database (keywords: ‘*endoglucanase*’, ‘*cellobiosidase*’, ‘*nitrate reductase*’, ‘*nitrite reductase*’, and ‘*nitrogenase*’, all with the additional query ‘*reviewed:true*’) and aligned to MAGs using diamond blastx [71] with default settings. A score was assigned to each MAG by calculating the fraction of queries matched. For example, when one out of two cellobiosidases from Uniprot gave a significant match, a score of 0.5 was assigned. All scores are given in Supplementary Table 1, Sheet 5. For Fig. 5b, a metabolic function was assigned when the score was greater than 0.1. When two MAGs with the same CAT annotation occurred in multiple communities, but the metabolic scores were not identical due to differences in genomic coverage, the highest score was used.

### Measuring the DNA concentration of MGE cocktails

To measure the DNA concentration present in DNA cocktails, compost mesocosms were established using the protocol described in Quistad et al. Briefly, samples were obtained from a compost heap (Plön, Germany) in September 2021. Five grams from each compost sample was transferred into a 100 ml glass flask containing a four cm^2^ piece of cellulose paper as a complex carbon source (Whatman cellulose filter paper) in 20 mL minimal M9 medium which contains 0.935 mM ammonium chloride. Subsequently, the founder mesocosms were incubated without shaking at 28 °C for 2 weeks. The lids remained slightly opened, allowing gas exchange.

After a 2-week incubation period, the cellulose paper was transferred into a falcon tube with 20 mL of minimal M9 medium and vortexed into a slurry. The slurries were vortexed, and 12 mL of cellulose slurry was centrifuged, for 10 min, at 4000 *g*. Subsequently, 10 mL supernatant was filtered through a 0.2 µm filter to produce the MGE cocktail. For DNA extraction, MGE cocktails were concentrated using an ultracentrifuge at ~26,500 g for 45 min. The supernatant was discarded and the pellet resuspended in 2 mL medium. DNA was extracted using the Phage DNA Isolation kit (Norgen Biotek Corp) or QIAprep Spin Miniprep Kit, following the manufacturer’s protocol. The amount of extracted DNA was assessed by Qubit HS assay. The measurements are found in Supplementary Table I, Sheet 4.

### Data analysis and visualisation

Data analysis and visualisation was done in R [78] v4.1.3, using the packages ggplot2 [79, p. 2], dplyr [80] v1.0.9, ggplotly [81] v3.4.1, patchwork [82] v1.1.1, gggenomes [83] v0.9.5.9000, ggraph (github.com/thomasp85/ggraph) v2.0.5, and rtracklayer [84] v1.60.0.

## Results

A bioinformatic pipeline was developed that identifies novel MGEs and other biologically relevant entities from metagenomic data. The pipeline does not rely on existing databases, but instead detects transfer of candidate MGEs based on sequences newly introduced into evolving communities. As such sequences are of ‘foreign’ nature, we refer to these as ‘xenotypic sequences’: the pipeline is termed xenoseq (https://github.com/bramvandijk88/xenoseq). For a full description of the bioinformatic pipeline see methods and Supplementary Material I and II.

Xenoseq was first benchmarked using simulated mock communities (Supplementary Materials II), and then applied to datasets from Quistad et al. The data by Quistad et al. is of particular interest, because the experimental design allows for dissemination of MGEs within *horizontal communities*, without allowing the migration of microbial cells (Fig. 1a). As short-read metagenome samples were prepared from all communities at various timepoints, it is possible to identify sequences that are not native to the evolved communities, that is, candidates for transfer of phages, plasmids, or other MGEs. However, not all sequences that newly appear are due to horizontal transfer of the nanobiome between allopatric communities. False positives can emerge when a sequence increases in abundance within sympatric communities, which can happen with rare species that are initially below the metagenomic detection limit (Fig. 1b). This false positive signal due to demographic change ought to apply equally to *horizontal* and *vertical* communities. Hence, after identifying newly emerged ‘unique sequences’ (xenoseq_find, Fig. 1c), further evidence for transfer is sought by aligning these contigs against sequences from allopatric communities (xenoseq_link, Fig. 1d). Note that false positive can still occur when parallel communities are very similar in composition, however in the Quistad experiment communities are diverse in composition (see ref. [52]). Then, as a final step, read mapping provides further insight into the origin and dissemination of xenotypic contigs (see Fig. 1e).

Note that xenoseq can in principle be applied to datasets that deviate from this particular experimental design. While Quistad et al. use glass mesocosms, mice would equally suffice when one is interested in the evolution of gut microbiomes. The experimental design could reveal selection pressures experienced by such natural communities, where the movement of MGEs (and the genes they carry as cargo) reflects what traits are relevant for community function under those conditions. Additionally, one could expose evolving communities to particular selection pressures, e.g. for antimicrobial resistance, to discover new MGEs that encode traits relevant under that condition. The only requirement is that longitudinal samples are taken from parallel communities which undergo exchange of MGEs or other biologically relevant entities.

### Horizontal compost mesocosms are enriched in xenotypic sequences

**Fig. 2:**
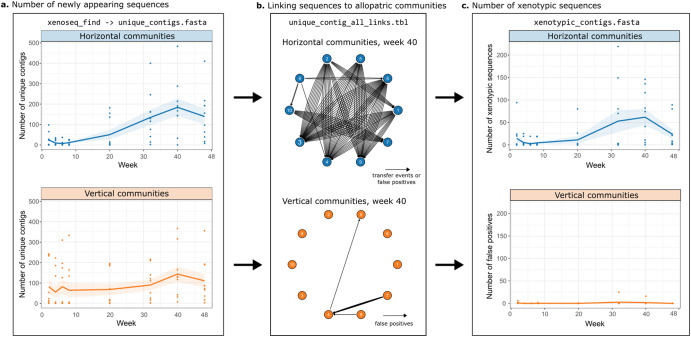
Horizontal communities are enriched in xenotypic sequences. **a** Unique contigs within horizontal (blue) and vertical (orange) communities identified by *xenoseq_find*. The solid lines represent the mean across 10 communities, shaded areas represents the standard error. **b** Aligning contigs identified by *xenoseq_find* to ancestral samples allows linking of donor-acceptor pairs (example network shown for horizontal and vertical communities at week 40, also see Supplementary Figs. 1+2). For horizontal communities, links in the network represent candidate elements that are horizontally transferred. For vertical communities, the links instead represent false positives, as transfer from allopatric communities is excluded by the experimental design. **c** The number of xenotypic contigs (sequences linked to at least one donor community) identified in horizontal communities is shown in blue, and false positives observed in vertical communities is shown in orange. Dots represent 10 different replicate communities sampled at each time point, the solid lines represent the mean a cross communities, and the shaded area represents the standard error.

The mesocosms from the study by Quistad et al. represent a ‘challenging case’ for the xenoseq pipeline, because of the unprecedented diversity and abundance of rare types. As mentioned above, shifts in abundance of rare types (as observed by Quistad et al.) can generate false positives (Fig. 1b). As a control, we therefore ran xenoseq on vertical communities, which are ‘closed’ and unaffected by movement of MGEs from allopatric communities. Indeed, we found that unique sequences are present in both horizontal and vertical communities (Fig. 2a, a total of 5617 and 6883 sequences respectively). However, only horizontal communities contained unique contigs that could be linked to allopatric communities (Fig. 2b). In total, 1756 (31.2%) of contigs in horizontal communities could be linked to an allopatric ‘donor’ community, versus only 58 (0.8%) of contigs from vertical communities. The remaining 58 contigs may be spuriously linked to allopatric communities due to overlap between ancestral compost communities. While all derived compost communities showed evidence of DNA sequences that were amplified over time, evidence for allopatric origins of these sequences was found exclusively in horizontal communities.

A possible confounding factor in the detection of xenotypic sequences arises if naked DNA included in the MGE cocktail were to remain intact during the 14-day incubation period thus ending up in the metagenomic data. To test this, we added exogeneous gDNA from *Escherichia coli* to the communities and monitored its persistence over time (see Methods). In the presence of the microbial communities, *E. coli* gDNA was no longer detectable within 48 h after addition (Supplementary Fig. 3), while it remained detectable throughout the 14-day experiment in community-free controls. Furthermore, with DNA concentrations of 0.67 ng/ml in the initial MGE cocktails, we consider it highly unlikely that these sequences would significantly contribute to the community metagenome samples after 14 days. Taken together, these data demonstrate that in order for xenoseq to predict horizontal transmission, DNA sequences must first persist degradation, and then be sufficiently amplified within the recipient community. Such amplification indicates some form of selection for the candidate MGE, e.g. because it is a selfish element or conveys a substantial fitness benefit to a host.

### Xenotypic sequences are enriched for phages and IS-elements

**Fig. 3:**
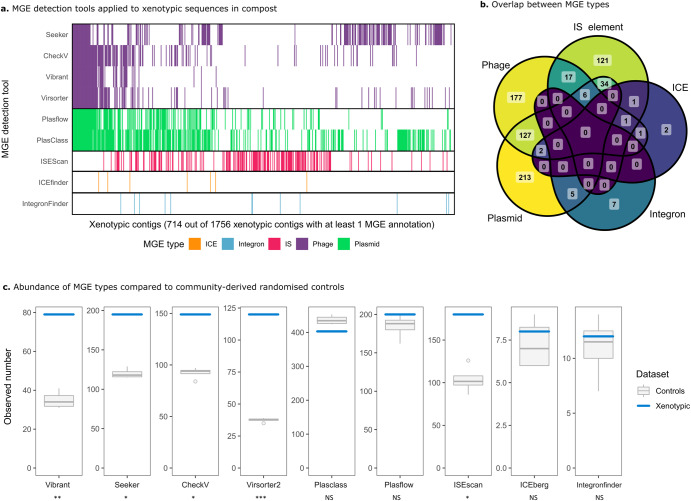
MGE detection tools reveal a diverse set of mobile elements in compost communities. **a** Various tools were used to predict MGEs including phages, plasmids, IS-elements, ICEs, and integrons in xenotypic sequences. On the *x*-axis are 714/1756 candidate MGE contigs with at least one MGE prediction. MGE tools are depicted on the *y*-axis. Coloured bars indicate that the contig was predicted by the corresponding tool. **b** A Venn diagram shows substantial overlap between MGEs, especially phages and plasmids. This may indicate interactions or hybridisation of phages and plasmids, but could also be the result of erroneous assignment by one of the tools. **c** The number of MGE annotations derived from xenotypic sequences is compared with four controls. Controls consist of contigs with similar lengths to the xenotypic contigs, but are sampled from the whole compost community metagenome. A modified T-test (Crawford-Howell [110]) was used to infer whether phages, plasmids, IS-elements, ICEs, or Integrons were over- or under-represented among xenotypic sequences (* = *p* value < 0.05, ** = *p* value < 0.01, *** = *p* value < 0.001). See precise p-values, including those corrected for multiple testing using a Bonferroni adjustment, in Supplementary Table 1, sheet 3.

The MGE cocktail that was distributed among mesocosms could contain bacteriophages, plasmids, naked DNA, membrane vesicles, and potential other (unknown) vehicles of transfer. The 1756 xenotypic sequences were interrogated using a variety of MGE detection tools, which provided predictions as to whether the sequences are viral [44–46, 74], plasmid^45,46^, IS-carrying^49^, ICE-carrying^47^, or integron-carrying^48^. We found that 714 (40.6%) of xenotypic sequences were identified by least one MGE prediction tool (see Fig. 3a). Considerable overlap was found between phages and plasmids (Fig. 3b), but also between other elements. These results indicate the existence of recombined or hybrid MGEs like phage-plasmids^29^. They may also highlight challenges of unambiguously predicting the MGE type from sequence using the selected tools.

To test whether xenotypic sequences are enriched in MGEs, these data were compared to an arbitrary set of sequences with a similar length distribution, sampled from the metagenomic contigs. Note that these community samples are still expected to contain many MGEs, but perhaps less than the xenotypic sequences. Indeed, phages and IS-elements appear to be over-represented among xenotypic sequences, whereas the numbers of plasmids, ICEs, and integrons are not significantly different (Fig. 3c). When corrected for multiple testing, phages remained significantly overrepresented but this did not hold for IS elements (see Supplementary Table I, Sheet 3 for all *p* values). In summary, because canonically selfish elements appear enriched, the results may further corroborate the importance of sequence amplification after introduction into a new community.

Interestingly, after applying nine state-of-the-art tools for predicting five categories of MGEs, 1042 xenotypic contigs (59.4%) remained unidentified.

### Movement of xenotypic sequences between communities

**Fig. 4:**
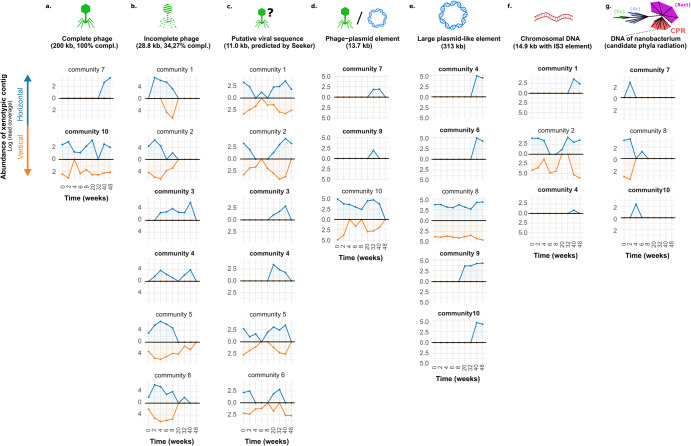
Tracing selected xenotypic sequences across communities. Results of *xenoseq_trace* for seven xenotypic sequences; **a** a phage with 100% read coverage as reported by CheckV (abbreviated as compl), **b** an incomplete yet highly abundant phage sequence (at certain time points covered by >10^6^ reads), **c** a putative viral sequence exclusively predicted by seeker, **d** a sequence reported to be both phage and plasmid by all relevant MGE tools, **e** a large plasmid that successfully establishes in four communities, **f** a putative chromosomal region flanked by a transposable element, and **g** a sequence not predicted to be an MGE, but annotated by CAT as being *Candidatus Saccharibacterium*. For all panels, abundances (y axes, average read coverage) are shown across communities over all time points (*x* axes). Abundance in horizontal communities is shown in blue, whereas abundance in vertical communities is shown on the opposing axes in in orange. Communities in which sequence are unique to the horizontal regime are shown in bold. Communities in which the contig was not observed are omitted for clarity. The full interactive data set is available as Supplementary Material.

To investigate the dissemination and dynamics of xenotypic contigs, xenoseq was used to map reads from all communities to the xenotypic contigs representing candidate MGEs. Figure 4 shows the results for seven selected examples: a complete 200 kb phage (Fig. 4a), an incomplete but abundant phage sequence (Fig. 4b), a putative viral sequence exclusively predicted by Seeker (Fig. 4c), an element predicted as both phage and plasmid unanimously (Fig. 4d), a large 313 kb plasmid (Fig. 4e), and a stretch of apparent chromosomal DNA adjacent to an IS3 element (Fig. 4f).

Xenoseq also revealed transfer of chromosomal bacterial DNA fragments lacking MGE predictions, many of which were annotated as *Candidatus Saccharibacterium* (Fig. 4g), a member of the candidate phylum radiation (CPR) bacteria first observed in human oral cavities [85]. Although this nanobacterium could indeed be sufficiently small to pass through the 0.2 µm filter, members of this species appear in both horizontal communities (community 1, 3, 4, 5, 7, 8, 9, and 10) as well as vertical communities (community 1, 3, 4, 7, 8, 9, 10), with varying estimates of genome completion (see Supplementary Table 1). The best representative MAG of this species was observed in HC10, which has a small genome (818 kb) that was nevertheless estimated to be 97% complete, and indeed encoded many of the relevant housekeeping genes (e.g. gyrA, recA, polA, topA, rpoB, and a single 16S ribosomal RNA gene). Comparing the 16S sequences with those from recently published CPR bacteria and DPANN archaea from groundwater samples [73], shows it clusters together with other CPR bacteria annotated as *Candidatus Saccharibacterium* (see Supplementary Fig. 4). Interestingly, alignment-free estimates of average nucleotide identity (fastANI v1.33) revealed that the (near-complete) nanobacterial MAGs from the communities are highly similar (up to 99.4% ANI). However, fastANI with respect the groundwater CPR bacteria yielded no results, which occurs when the genomes compared are too dissimilar. Taken together, these analyses suggests that the nanobacterium in our compost mesocosms are closely related to, but distinct from, these previously published bacterial MAGs.

Co-occurrence analysis (see Supplementary Fig. 5a) further reveals a potential connection between the identified nanobacterium and the genus *Cellvibrio*. Tracking the abundance of both players over times suggests a potential boom-and-bust dynamic, punctuated by and long periods of stasis (Supplementary Fig. 5b). Such dynamics may be indicative of a pathogenic lifestyle.

### Compost mesocosms form distinct community types

Thus far, we have illustrated that treating evolving microbial communities with an ‘MGE cocktail’ derived from all communities promotes the movement of various MGEs, and even nanobacteria between communities. We have also shown that xenotypic contigs are especially enriched in SGEs, suggesting the importance of independent sequence amplification for the survival of MGEs after introduction into allopatric communities. In the following sections, we examine the ecological and evolutionary consequences of this treatment. To investigate whether and how the horizontal communities are distinct from the vertical communities, we studied the abundance of MAGs. These MAGs were assigned a taxonomic classification using the Bin Annotation Tool (BAT). When taxonomic rank could not be determined reliably due to conflicting ORFs, a higher-order taxonomic rank was assigned instead, ensuring all MAGs had a robust classification.

**Fig. 5:**
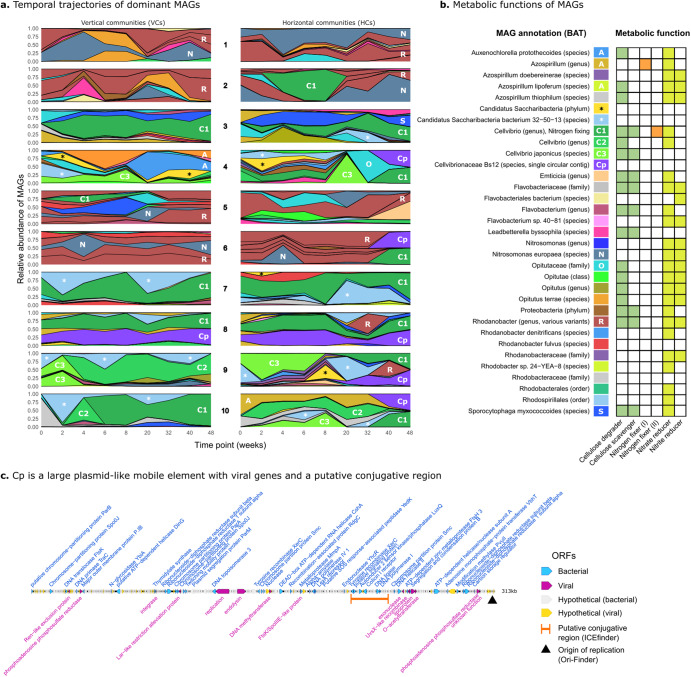
Horizontal communities show repeated shifts in dominant MAGs. **a** For all 20 replicate experiments (10 horizontal, 20 vertical), abundance (relative read coverage) is shown for MAGs over time. For clarity, only MAGs that are highly abundant across multiple time points are shown (representing on average 59.6% of the total community), and abundance is given as the fraction of reads mapping only to these particular MAGs. A subset of MAGs are labelled with a letter (C1, R, N, Cp, etc.) corresponding to the legend in (**b**). **b** Metabolic functions assigned to each MAG. MAGs encoding putative endoglucanases, cellobiosidases, nitrogenases (I and II), and nitrate/nitrite reductase were assigned roles to cellulose degraders, cellulose scavengers, nitrogen fixers, nitrate reducers, and nitrite reducers, respectively (see Methods). A score was calculated based on the number of significant hits to these protein sequences, and MAGs with a score greater than 0.1 are coloured in the heatmap. **c** A circular, 313 kb mobile element (Cp) that emerges in four independent horizontal communities. The element contains both plasmid-like and phage-like traits.

Each MAG was screened for various genes related to two metabolically relevant functions: cellulose degradation and nitrogen metabolism (see Methods and Supplementary Table I, Sheet 5). The relative abundance of dominant MAGs is shown for each community in Fig. 5a (for an interactive graph of all MAGs, see Supplementary Files). The MAGs are dominated by either *Rhodanobacter* (shown in brown in Fig. 5a) or *Cellvibrio* lineages (shown in green). Members of both these genera are able to degrade cellulose, the sole carbon source in the mesocosms (Fig. 5b). As many other MAGs do not have this ability, *Rhodanobacter* and *Cellvibrio* appear to occupy a similar niche and are the primary degraders of cellulose, which could explain why they appear to be mutually exclusive. The two community types furthermore have distinct lineages that coinhabit the mesocosms. For example, the communities dominated by *Rhodanobacter* lineages often coexist with *Nitrosomonas europaeae* (steel blue), which can reduce nitrate and nitrite (Fig. 5b). Communities dominated by *Cellvibrio* instead frequently host species of above-mentioned *C. Saccharibacteria* (denoted by an asterisk in Fig. 5a, b.

### Horizontal communities show more changes in primary degraders

As can be seen from Fig. 5a, vertical communities (VCs, left) are relatively stable in the composition of dominant MAGs, establishing either with *Cellvibrio* or *Rhodanobacter* as primary cellulose degraders. Horizontal communities (HCs) however show rapid shifts in these primary degraders. For example, HC2 initially establishes with *Rhodanobacter* (brown) as the primary degrader. However, during week 4 to 8, a *Cellvibrio* lineage (C1, green) becomes more dominant relative to other MAGs. Finally, *Rhodanobacter* re-emerges as the primary degrader. *Vice versa*, HC8 and HC9 are initially established as *Cellvibrio*-communities, and show a transient appearance of a *Rhodanobacter* lineage after 32/40 weeks.

While the above-mentioned disruptions caused by the MGE cocktails are transient, *Cellvibrio* C1 in HC6 appears to completely overturn the ecosystem structure that was established during the preceding 32 weeks, although it is unclear whether this disruption is transient since we do not have measurements after 48 weeks. *Cellvibrio* C1 also emerges in three other mesocosms independently (in HC9 at week 8, and HC4 and HC10 at week 32), and its emergence is always accompanied by a MAG containing only a single 313 kb contig (shown in purple in Fig. 5a, henceforth called Cp). This contig is of circular nature, and is in fact the same plasmid-like element earlier identified by xenoseq (Fig. 4e). The large mobile element carries plasmid partitioning proteins (e.g. ParB and ParM) typically associated with low copy-number plasmids [86, 87] (see Fig. 5c), which is consistent with it co-occurring with *Cellvibrio C1* in an approximate 1:1 ratio. In addition to features that are akin to large plasmids, Cp also carries many ORFs associated with phages as evident in matches to the viral PHROGs-database^66^ (for example, integrase and endolysin). Finally, Cp carries a putative conjugative region identified by ICEfinder. As this plasmid is present in earlier time points only in VC/HC8, this suggests this community as the donor. We hypothesise that Cp transfers horizontally, enabling Cellvibrio C1 (the putative host) to displace the previously established ecosystem structure.

### Ammonia production appears elevated in Cp+ communities

Because cellulose is the only exogenously provided carbon source in the mesocosms, the ability to degrade cellulose is not surprisingly found in many MAGs (see Fig. 5b). While all the *Cellvibrio* lineages have the ability to degrade cellulose, one particular *Cellvibrio* (C1) is unique in also carrying a nitrogen fixing enzyme (flavoprotein, EC 1.19.6.1). The ability to fix nitrogen is likely important, because 1 mM ammonium chloride from the M9 medium added every 14 days is the only source of exogenously supplied nitrogen. We hypothesised that the Cp plasmid may have regulatory impacts on C1 host metabolism, perhaps favouring fixation of nitrogen.

**Fig. 6:**
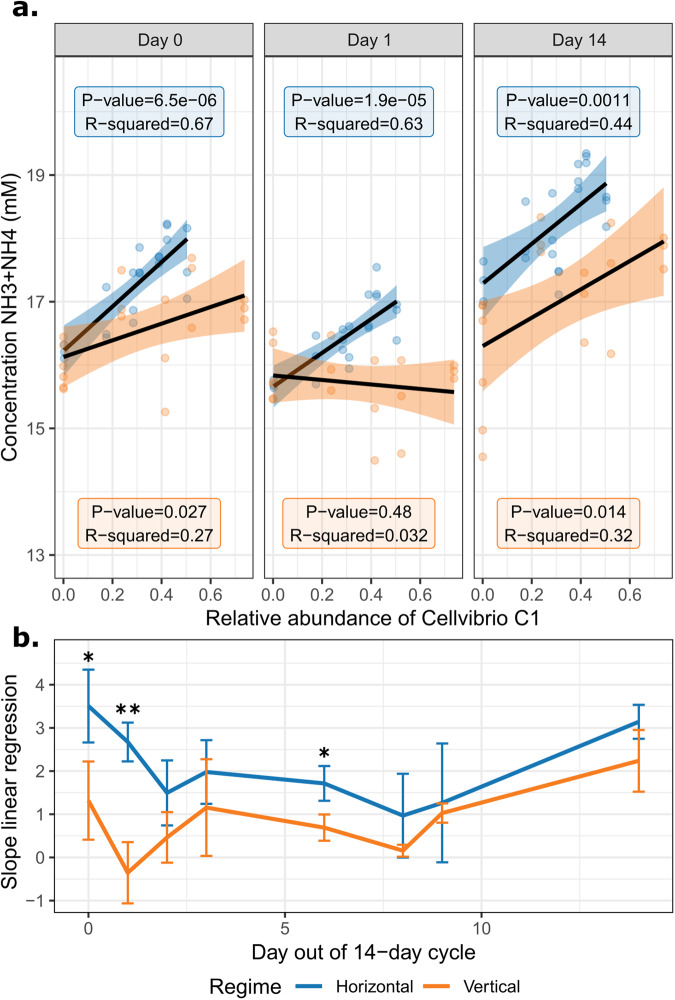
*Cellvibrio* C1 appears to fix more nitrogen in horizontal (Cp+) communities. **a** The relative abundance of *Cellvibrio* C1 was determined by the proportion of reads from the sample at week 48 mapping against this MAG, which was then plotted against ammonia production measurements from Quistad et al [52]. *Cellvibrio* C1 was present in five vertical communities and seven horizontal communities, yielding a total of 12 abundance values for *Cellvibrio C1*, plotted on the x-axis. For each of these values, three technical replicate measurements of ammonia were performed at several time points in the 14-day cycle. The y-axis shows the measured ammonia concentrations (NH3 + NH4). A linear regression model was fitted to the data points. For day 0, 1 and 14, these fits are shown with their corresponding *p* values and *R*^2^ values (see Supplementary Fig. 6 for all days). **b** The slope of the linear regression is plotted for all days in the bottom panel, revealing that horizontal communities consistently show a steeper slope, suggesting that the large Cp plasmid promotes nitrogen-fixation by the *Cellvibiro* C1 MAG. Error bars denote the standard deviation of the slope across three technical replicates of ammonia production measurements. The asterisks indicate significant differences (Student’s T test) between the horizontal and vertical communities at that time point (* = *p* value < 0.05, ** = *p* value < 0.01).

In the study by Quistad et al., ammonia production was measured at the end of the experiment (*T* = 48 weeks). By plotting the abundance of *Cellvibrio* C1 against data on ammonia production, a strong positive correlation was observed (Fig. 6a) in horizontal communities, but much less so in vertical communities. Note that C1 in most horizontal communities carries the Cp plasmid, and C1 in vertical communities does not. We suggest that this indicates that C1 may be responsible for the accumulation of ammonia, but only in the presence of the Cp plasmid. Indeed, by taking the slope of the linear regression, we found that horizontal communities show a significantly steeper correlation between C1 and ammonia production rates, especially early in the growth cycle (Fig. 6b). Finally, when focussing only on horizontal communities where Cp was absent (HC3 and HC7), no positive correlation was observed (Supplementary Fig. 6). These data suggest that Cp promotes the fixation of nitrogen by *Cellvibrio C1*.

## Discussion

MGEs are important determinants of microbial evolution with far-reaching effects within the context of communities. As diverse as microbiomes are, the nanobiome—the set of Darwinian entities dependent on microbial hosts—is likely even more diverse. We developed a bioinformatic pipeline called xenoseq to shine light on the nanobiome, inspired by experiments from Quistad et al. Using time-resolved metagenomic data, xenoseq can distinguish between two sources of novel sequences, (i) those that arise due to local demographic changes, and (ii) those that are the consequence of horizontal transmission of nanoscale entities between parallel communities. We show that the latter category of *xenotypic sequences* requires amplification after introduction into allopatric communities. We found that xenotypic sequences were especially enriched in selfish genetic elements (phages and IS elements). While other MGEs such as plasmids and ICEs were not enriched, we did find some unanticipated players to transfer horizontally via the filtrate, including a 313 kb plasmid and a CPR nanobacterium. Taken together, our data shows that the pipeline can successfully identify a broad range of interesting MGEs, without any prior knowledge as to their identity.

The experimental strategy of Quistad et al. and our bioinformatic pipeline can be applied to any microbial community and can accommodate specific selection pressures, such as antimicrobial resistance [88, 89], heavy metal resistance [90], or bioremediation capacity [91]. Furthermore, even without any evident selection pressures, the method can provide direct evidence of selection pressures experienced by communities, through identification of traits transferred horizontally. Quistad et al. observed the enrichment of nitrogen metabolism genes in horizontal communities, which in this study, we have been able to link to the proliferation of a large 313 kb plasmid-like sequence. Given its apparent indirect role in nitrogen fixation, we hypothesise that amplification of this element confers a fitness advantage (Fig. 6). The mechanism of horizontal transfer is unknown, but is unlikely to be via naked DNA, leading to the possibility that the element maybe packaged inside membrane vesicles (MVs) [92–95], as recently shown in *Klebsiella pneumoniae* [96].

Besides the movement of MGEs, our study also suggests competition between ecologically relevant species is enhanced in horizontal communities. This enhanced competition may be due to ‘kill the winner’ dynamics [97], whereby phages preferentially reduce the abundance of established microbial species, while at the same time improving their own evolutionary fate, making niche space available for competitors. A similar dynamic was observed in a recent study that illustrates how the induction of prophages can significantly impact the assembly of chitin-degrading communities [98]. Notably, these shifts in abundances could exacerbate the problem with false positives as we observed in this study. Taken together, we argue that it is important to improve understanding of how MGEs influence the competition between microbial species—for example in societally relevant systems such as the human gut [99] or plant rhizospheres [100, 101]—which may furthermore enable us to make better evolutionary predictions in the future [102].

An unexpected finding of our study was the apparent transmission of a CPR nanobacterium of the phylum *Saccharibacteria*. Indeed, close relatives of this organism are not much larger than a phage^75^, and the reduced genome (~818 kb) is indicative of a symbiotic relationship with another species. The nanobacterium was shown to cluster closely with CPR bacteria observed in groundwater ecosystems [73], where many ultrasmall cells are shown to stick to the cell surface of larger bacteria. Similar patterns have been observed for*Vampirococcus lugossi*, which are also thought to be epibionts that attach to photosynthetic bacteria [103]. Rather than episymbionts, however, we suggest that the nanobacterium in our study may be an intracellular parasite, as the boom-and-bust dynamics shown punctuated by and long periods of stasis shown in Supplementary Fig. 5b are reminiscent of predatory dynamics as observed for *Bdellovibrio bacteriovorus*. Our co-occurrence analysis (see Supplementary Fig. 5a) revealed a potential connection with a bacterial lineage belonging to the genus *Cellvibrio*. Identification of this nanobacterium, and the newly generated hypothesis of a host-parasite relationship, highlight an additional power to our approach. Excitingly, many species of *Cellvibrio* can be isolated and cultured, paving the way for future studies on the nanobacterium and its interactions with the host.

A number of annotation-free methods to track HGT in microbial ecosystems have recently been developed, relying for example on differential read coverage [104], assembly graphs [105, 106], discordant read pair mapping [107], or Hi-C metagenomics [88, 108, 109]. However, compared to our approach, these methods do not provide insights into the dynamics and functional consequences of MGE transmission, and how this flux of DNA shapes microbial communities. Our strategy combines experimental intervention, metagenomics and bioinformatic analysis to discover elements capable of transmission as nanoparticles and subsequent amplification, without prior knowledge of the elements themselves. In other words, using our method, it becomes possible to interrogate the nanobiome free from pre-conceived notions of the identity of its members.

While we illustrate the discovery of novel and interesting biological entities, there is also considerable potential for false positives and false negatives. As partially addressed in this study, metagenomic detection limits make it extremely difficult to say for certain whether a sequence was ancestrally present in a community, or whether it was acquired from an allopatric community. Moreover, it is possible that treating communities with cocktails of MGEs has indirect impacts on the community composition, which if resulting in rare species becoming more abundant, would further exacerbate this problem. While our pipeline tries to reduce these problems by linking sequences to their community of origin, this strategy trades off with false negatives, as genuine horizontal gene transfer events may be erroneously discarded. While we argue that advancements like Hi-C metagenomics may help to alleviate some of these difficulties, we also suggest that future studies apply the experimental protocol to simpler (synthetic) communities, making it easier to distinguishing foreign DNA from rare sequences. Note however, that such simplification comes with its own trade off, as it hampers our ability to study the processes that are inherent to complexity itself [38]. We therefore argue that in order to further our understanding of complex microbiomes, it is also important to embrace complexity—with all the potential sources of noise that entails—and identify the simple rules that drive the eco-evolutionary dynamics of microbial communities.

### Concluding remarks

Our work confirms earlier conclusions that microbial ecosystems are greatly influenced by the flux of DNA created by MGEs, even when these fluxes are initially subtle. Hence, we argue that understanding the nanobiome—the zoo of Darwinian entities much smaller than bacteria—is crucial to understanding microbial ecology, and how these systems eventually scale up to impact entire ecosystems.

### Supplementary information

Supplementary materials

## Data Availability

Raw sequencing data are taken from Quistad et al., and are publicly available online (https://www.mg-rast.org/mgmain.html?mgpage=project&project=mgp18485). MAGs, interactive datasets, and R scripts for designing the mock communities are published on Zenodo (10.5281/zenodo.7589193). Any further requests for data can be sent to rainey@evolbio.mpg.de.
